# Daily activity during stability and exacerbation of chronic obstructive pulmonary disease

**DOI:** 10.1186/1471-2466-14-98

**Published:** 2014-06-02

**Authors:** Ayedh D Alahmari, Anant RC Patel, Beverly S Kowlessar, Alex J Mackay, Richa Singh, Jadwiga A Wedzicha, Gavin C Donaldson

**Affiliations:** 1 Centre for Respiratory Medicine, University College London, Royal Free Campus, London, UK

**Keywords:** COPD, Exacerbation, Daily step-count, Physical activity, Daily monitoring

## Abstract

**Background:**

During most COPD exacerbations, patients continue to live in the community but there is little information on changes in activity during exacerbations due to the difficulties of obtaining recent, prospective baseline data.

**Methods:**

Patients recorded on daily diary cards any worsening in respiratory symptoms, peak expiratory flow (PEF) and the number of steps taken per day measured with a Yamax Digi-walker pedometer. Exacerbations were defined by increased respiratory symptoms and the number of exacerbations experienced in the 12 months preceding the recording of daily step count used to divide patients into frequent (> = 2/year) or infrequent exacerbators.

**Results:**

The 73 COPD patients (88% male) had a mean (±SD) age 71(±8) years and FEV_1_ 53(±16)% predicted. They recorded pedometer data on a median 198 days (IQR 134–353). At exacerbation onset, symptom count rose by 1.9(±1.3) and PEF fell by 7(±13) l/min. Mean daily step count fell from 4154(±2586) steps/day during a preceding baseline week to 3673(±2258) step/day during the initial 7 days of exacerbation (p = 0.045). Patients with larger falls in activity at exacerbation took longer to recover to stable level (rho = −0.56; p < 0.001). Recovery in daily step count was faster (median 3.5 days) than for exacerbation symptoms (median 11 days; p < 0.001). Recovery in step count was also faster in untreated compared to treated exacerbation (p = 0.030).

Daily step count fell faster over time in the 40 frequent exacerbators, by 708 steps/year, compared to 338 steps/year in 33 infrequent exacerbators (p = 0.002).

**Conclusions:**

COPD exacerbations reduced physical activity and frequent exacerbations accelerate decline in activity over time.

## Background

Chronic obstructive pulmonary disease (COPD) is a global cause of morbidity and mortality and an impairment of health status [1]. Patients with COPD experience episodes of acute worsening of respiratory symptoms termed exacerbations, often triggered by infections [1]. Frequent exacerbations are a stable feature of the disease [2] and have important impacts, such as, accelerating decline in lung function, reducing quality of life and impose higher health care utilization and costs [3-5]. Respiratory symptoms following an exacerbation can take a number of weeks to return to baseline [6]. COPD patients are less likely to go outside during an exacerbation [7]. Also, patients with frequent exacerbations experience a significant faster decline in the amount of time spent outdoors [7]. Depression is a significant comorbid condition in COPD patients [8] and associated with increased risk of exacerbation and hospitalisation [9].

COPD although primarily affecting the respiratory system is known to have extra pulmonary effects such reduced patient activity and skeletal muscle dysfunction. Studies using accelerometers have shown that activity declines at exacerbation especially in hospitalized patients in comparison to a month later [10,11] and over time [12]. Low levels of physical activity assessed with a multisensory arm-band (SenseWear) is strongly associated with all-cause mortality in patients with COPD [13] and data from an ankle-worn accelerator independently predicts exacerbation frequency [14].

Most clinical trials of early post-exacerbation pulmonary rehabilitation (PR) trials have shown significant improvement in exercise capacity, skeletal muscle strength, dyspnoea, quality of life and prevent de-conditioning [15-17]. Despite this evidence, patients treated in the community for an exacerbation are not actively encouraged to maintain their physical activity during exacerbation recovery. Possibly, information is lacking concerning the extent that physical activity decreases during these non-hospitalized events. These data would be needed for determining the sample size required for a clinical trial of early PR in community treated exacerbations.

Prospective monitoring of physical activity is necessary for quantifying the effects of exacerbation on activity as baseline measurements falls over time and may vary with season. Accelerometer-based monitoring devices are expensive and require regular clinic visits to download the data and are therefore not ideal for the long-term use required to prospectively capture relatively rare events such as exacerbations. Accelerometers may slightly under-estimate step count at the slow walking speeds expected in COPD patients [18]. Pedometers are cheap and simple to use, but there is little published data to support their use in COPD patients. The aims of this study were to prospectively evaluate daily step-count determined with a simple pedometer, before and during the onset and recovery of an exacerbation. We have also evaluated the longitudinal trend of daily activity in patients with a history of frequent and infrequent exacerbations.

## Methods

### Patient recruitment and characteristics

Seventy three patients were recruited from the London COPD cohort. These patients recorded daily pedometry data for a minimum of 35 days with the initial 7 days considered as training and discarded for the purposes of analysis. The study took place over 19 months between April 2011 and November 2012.

All 199 patients in our rolling cohort were considered for participation in this study. 24 patients were not eligible as they used a walking support (cane or frame), were confined to a wheel chair or used ambulatory oxygen cylinders; 30 refused. We eventually provided pedometers to 145 patients. Data was successfully acquired from only 73 patients due to the following reasons a) 21 patients once issued refused to use the pedometer b) 19 patients lost their pedometers c) 23 patients recorded less than 35 days of data whilst stable d) 9 pedometers malfunctioned.

COPD was defined as a post-bronchodilator forced expiratory volume in one second (FEV_1_) to forced vital capacity (FVC) ratio below 0.70 and FEV_1_ expressed as a percentage of predicted FEV_1_ of less than 80%. Patients were categorised as moderate, severe or very severe according to the Global Initiative for Chronic Obstructive Lung Disease (GOLD) classification [19]. Patients with a history of another significant respiratory disease or considered unable to complete daily diary cards excluded [4]. Patients were recruited when clinically stable, at least six weeks after their last exacerbation.

At recruitment, age, gender, chronic respiratory symptoms and smoking history were noted, and height and weight measured. FEV_1_ and FVC were measured with a Vitalograph Gold Standard spirometer (Vitalograph Ltd, Maids Moreton, UK).

### Daily monitoring

All patients were asked to complete a daily diary card on which they recorded their morning post-medication peak expiratory flow (PEF) measured with a mini-Wright peak-flow meter (Clement-Clarke International Ltd, Harlow, UK). They also recorded any worsening in their respiratory symptoms above normal and the number of hours spent outside their home.

Patients were also instructed to wear a Yamax Digi-walker SW-200 pedometer on the left side of their body [20,21] all the time, except when sleeping or showering. Pedometer placement was standardized by placing it on the belt or waistband, in the midline of the thigh, consistent with the manufacturer’s recommendation [22]. This pedometer has been shown to accurately measure steps in free-living individuals [22] and in normal and moderately obese patients and [23] detected differences in physical activity of COPD patients recorded their daily step count on the diary cards.

### Exacerbation definition

An exacerbation was defined as an increase for two consecutive days in respiratory symptoms, with at least one major symptom (dyspnoea, sputum purulence or sputum volume) plus either another major or a minor symptom (wheeze, cold, sore throat, and cough) [6] .Five consecutive symptom free days were required before identification of the next exacerbation. Symptoms were disregarded in identifying exacerbation onset if recorded continuously in the preceding 5 days [24].A small proportion of exacerbations for which no diary-card symptoms had been recorded by questioning the patient at clinic visits about any recent prescriptions [24] Symptom counts were obtained by summating each increased respiratory symptom recorded on diary cards per day.

Patients were then divided into two groups, based on the number of exacerbations in the 12 months preceding the start of the study, those with 2 or more exacerbations per year were called frequent exacerbators and those with 0 or 1 exacerbation per year called infrequent exacerbators [25].

Changes at exacerbations in daily step-count, symptoms count, PEF and hours spent outdoors were assessed by comparison of the average value over a 7 day baseline period which started 2 weeks before onset with the average value over a 7 days exacerbation period starting on the day of exacerbation onset. Recovery was determined as the day after exacerbation onset when a 3 day moving average of a parameter matched or exceeded its baseline value. A moving average was used to avoid false early recoveries when step count or lung function improved for just a single day, but then remained below baseline for a few more days [6].

### Ethics

The study was approved by the London-Hampstead research ethics committee and all patients gave written informed consent (REC 09/H0720/8). The current study is entirely novel and has not been reported before except in abstract form (*European Respiratory Society Annual Congress 2013*, *Barcelona*, *Spain 7*–*11 September*).

### Statistical analysis

Data were analyzed with STATA 8.2 (Stata Corporation, College Station, TX) and PASW statistics V.21 (SPSS Inc.). Normally distributed data are reported as a mean and standard deviation (SD) or standard error of the mean (SEM) and skewed data reported with a median and inter quartile range (IQR). Comparisons were made by paired Student t test or Wilcoxon signed-rank test as appropriate. Stable mean daily step count and other patient characteristics were related with a Pearson correlation or Spearman rank correlation. Random effect linear regression models were used to assess annual decline in daily stable step count and whether the decline was faster in frequent than infrequent exacerbators. A stable step count was defined as outside a period starting 2 weeks before and ending 2 weeks after an exacerbation. Data from patients who experienced multiple exacerbations were averaged to avoid bias through repeated measures. However, we analyzed exacerbations as individual events when investigating whether the characteristics of exacerbations (respiratory symptoms, treatment, change in step count) was associated with a fall in activity or recovery. Significant was taken as p < 0.05.

## Results

The 73 COPD patients (51 male, 22 female) studied had moderate to very severe COPD (Table 1). Between 8^th^ April 2011 and 30^th^ November 2012, daily step count was recorded on 17,161 days with a median of 198 days per a patient (IQR 134–353; range 29 to 540) days per patient. There were no significant differences in the patient characteristics between the 73 patients involved in this study and 126 patients excluded for reasons mentioned in the methods (Table 1).

**Table 1:** Characteristics of the 73 COPD patients in the study and 126 COPD patients in the Cohort not recruited to the study

	**73 COPD patients**		**Remaining 126 COPD patients in the Cohort**		***P***-***value***
	***Mean***	**(± *SD *)**	***Mean***	**(± *SD *)**	
**Age (years)**	71.1	(±8.7)	70.2	(±8.8)	0.51
**FEV**_**1 **_**(l)**	1.31	(±0.5)	1.40	(±0.5)	0.25
**FEV**_**1 **_**(% predicted)**	52.9	(±16.5)	56.2	(±16.1)	0.22
**FVC (l)**	2.79	(±0.9)	2.76	(±0.9)	0.83
**FEV**_**1**_**/FVC (%)**	47.8	(±12.6)	50.7	(±12.3)	0.11
**BMI (kg/m**^**2**^**)**	26.8	(±5.6)	27.0	(±5.1)	
	***Median***	**( *IQR *)**	***Median***	**( *IQR *)**	
**Exacerbations/year**	2	(1.0-3.0)	1.4	(0.7-3.0)	0.40
	**%**				
**Sex (males)**	69.9		62.6		0.36
**Chronic bronchitis**	54.3		54.5		0.98
**Smoking at recruitment**	35.6		32.0		0.77

SD=Standard Deviation, IQR=Inter-quartile range.

### Decline of daily step-count between patients with frequent and infrequent exacerbations

**Figure 1:**
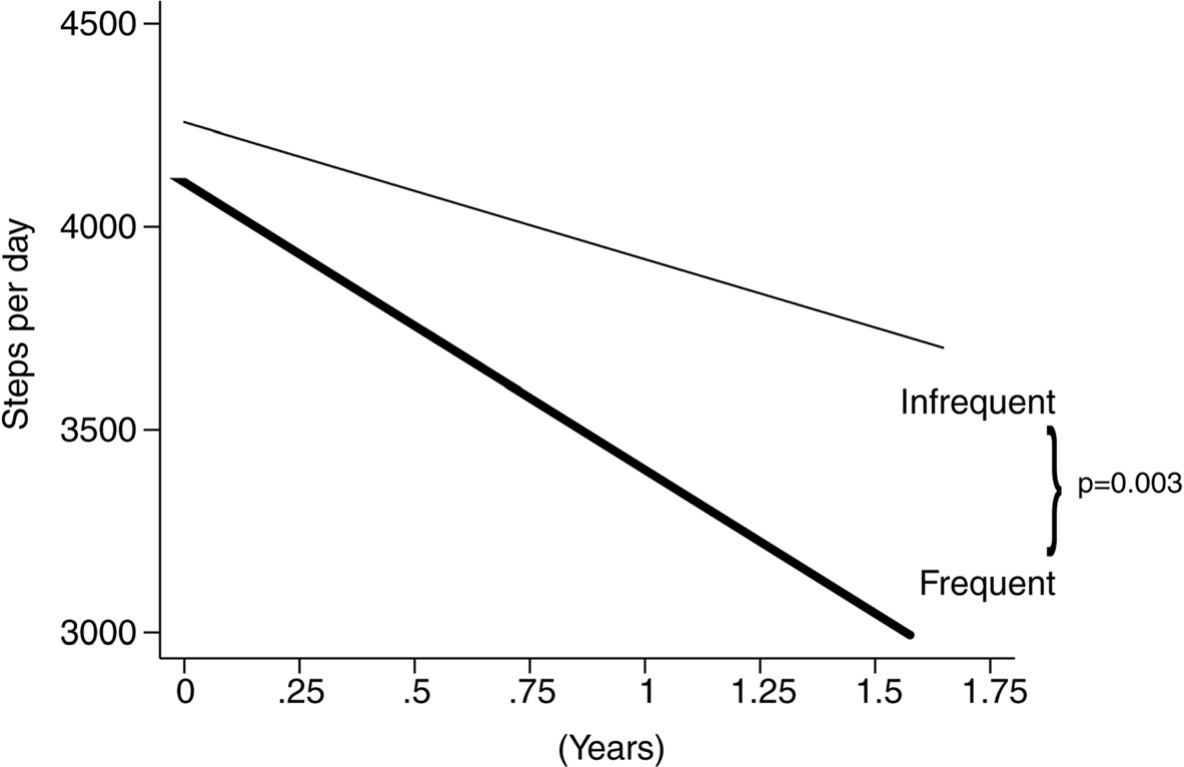
**Daily step**-**count of 33 infrequent (number of days with data** = **6878) and 40 frequent (number of days with data** **=** **7775) exacerbators; predicted values obtained from the random effects, linear regression model (test of interaction, p** **=** **0.002).** Time 0 corresponds to the start of the study.

Daily step-count was recorded in the stable state on 14,653 days (median 169 days per patient; IQR 113–285; range 29–488) and for 2508 days with exacerbation (median 21 days per patient; IQR 0–57; range 0–239). Separately, daily step-count fell in the 33 infrequent exacerbators by 338 steps/year [95% CI: −504 to −170] compared to 708 steps/year [95% CI: −867 to −549] in the 40 frequent exacerbators (both p < 0.001). The annual decline in daily step-count was significantly faster in the frequent exacerbators (p = 0.002; see Figure 1).

### Time course of daily step-count at COPD exacerbation

Thirty seven patients experienced 79 exacerbations and the characteristics of these patients are reported as additional file table (see Additional file 1: Table S1). The median time since the last exacerbation was 85.5 days (IQR 42–193). The shortest interval was 15 days. There was no record of a preceding exacerbation for 7 exacerbations as these patients had been recently recruited. There were no significant differences in patient characteristics between these 37 patients and the 36 who did not experience an exacerbation except a higher FVC (L).

**Figure 2:**
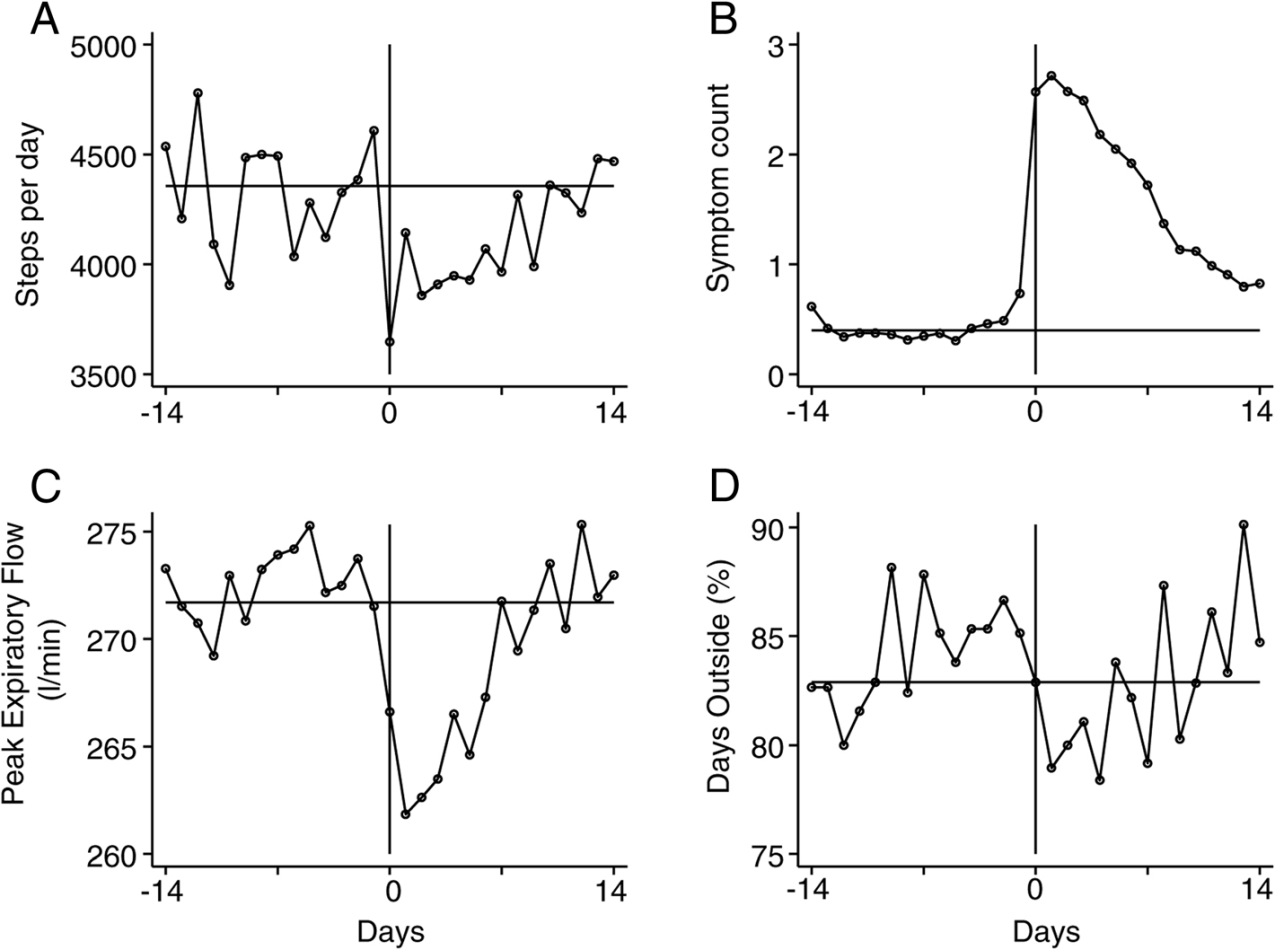
Time courses of daily step-count (A), symptoms (B), peak expiratory flow (C) and time spent outside (D) at COPD exacerbation.

Table 2 shows that daily step-count, symptoms count and PEF fell significantly at exacerbation but not hours spent outdoors or whether the patient went out or not. Daily step-count took a median 3.5 (IQR 1–8) days to return to baseline levels (see Figure 2A). Symptom count rose and took 11 (IQR 8–17) days to resolve (Figure 2B) and PEF (Figure 2C) took a median 4 (0–15) days to return to normal. Hours spent outdoors per day returned to baseline levels within 1.4 (IQR 0.3-5.3) days (Figure 2D). Recovery in step count was significantly earlier than symptoms (p < 0.001) but not PEF (p = 0.33) or hours spent outdoors (p = 0.18).

**Table 2:** **Comparison between baseline and first 7 days of exacerbation (mean** ± **SD)**

	**Stable**	**Exacerbation**	**Difference**	**p-value**
**Daily step-counts (step/day)**	4154 (±2586)	3673 (±2258)	−480 (±1408)	0.045
**Symptom count**	0.4 (±0.7)	2.4 (±1.0)	1.9 (±1.3)	<0.001
**Peak Expiratory Flow (L/Min)**	273 (±109)	266 (±108)	−7 (±13)	0.005
**Time outdoors (hours/day)**	3.4 (±1.8)	3.2 (±1.8)	−0.1 (±1.1)	0.51
**Percentage of days on which patient went outdoors (%)**	84.4 (24.2)	79.6 (26.1)	−4.8 (±18)	0.13

### The relationship between fall in daily steps–count and respiratory symptoms and activity recovery

**Figure 3:**
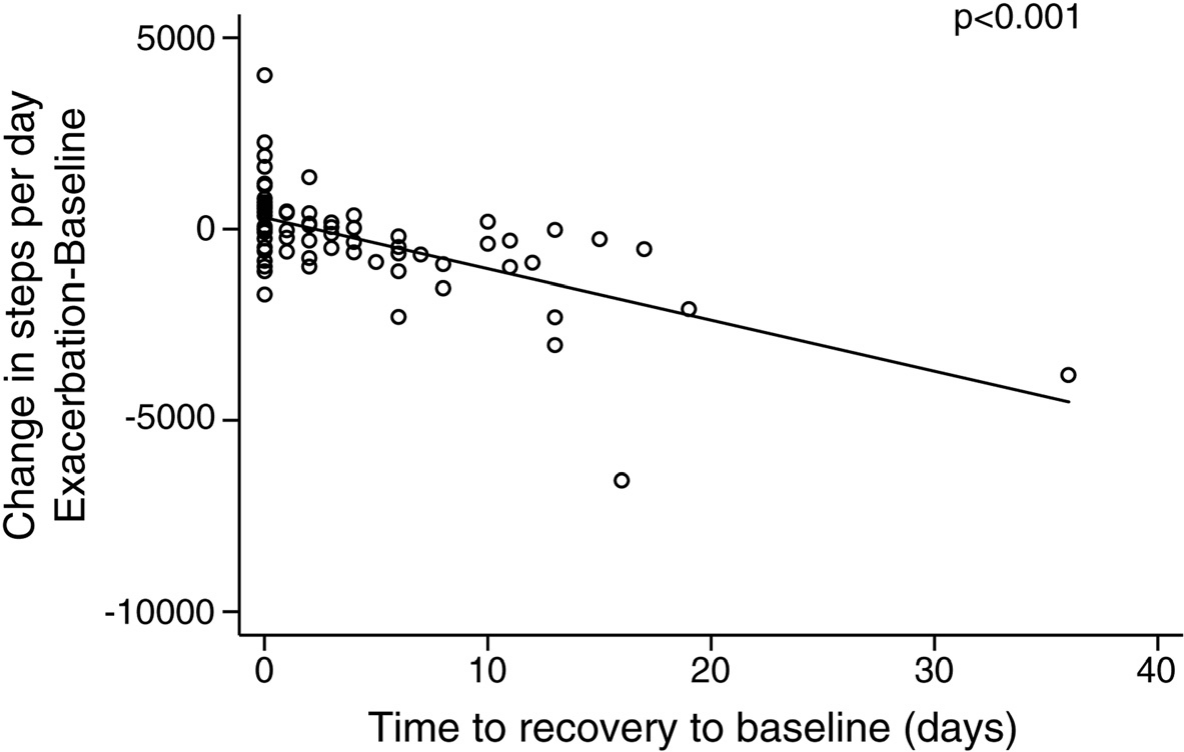
**Change in daily steps between baseline and exacerbation against time to recovery to baseline; 79 exacerbations (p** **<** **0.001).**

For the 79 exacerbations, those with the largest falls in daily step count took longer to recover to baseline (rho = −0.56; p < 0.001, Figure 3). During the 79 exacerbations, symptoms of dyspnoea were reported in 83.5%, sputum purulence in 43.0% and increase sputum volume in 67.1% as major symptoms. Minor symptoms of cold were reported in 43.0%, wheezing in 49.4%, sore throat in 21.5% and cough in 54.4%. No relationship was seen between symptoms and fall in daily step count at exacerbation or recovery in steps count with one exception the fall in step count and symptoms of a sore throat (p = 0.037).

### Activity and treatment

**Figure 4:**
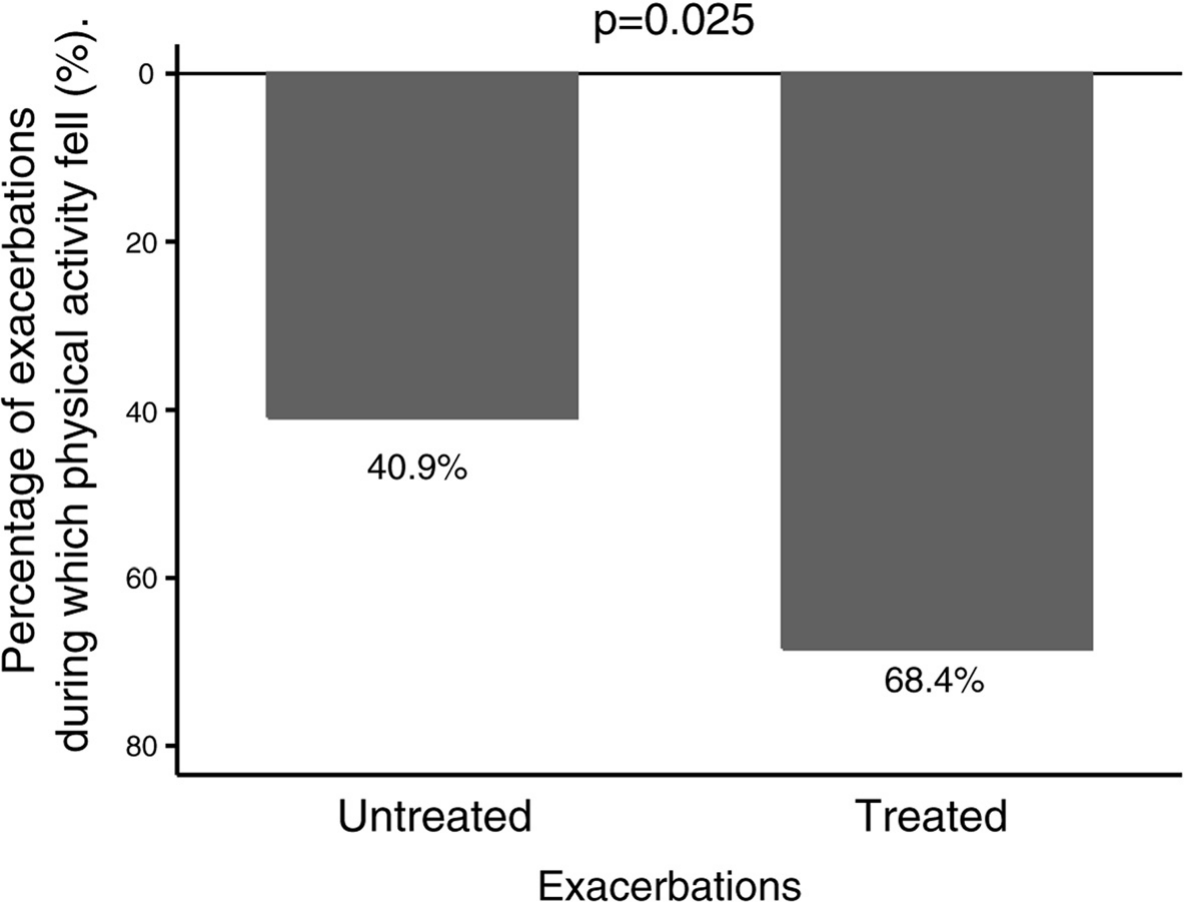
**The percentage of treated and untreated exacerbations that daily activity fell between baseline (average days −14 to −8) and exacerbation (average days 0 to 6).**

Fifty-seven exacerbations (11 antibiotics only; 7 oral corticosteroids only; 39 both antibiotics and oral steroids) were treated in the COPD clinic and 22 were untreated. Untreated exacerbations showed faster activity recovery (median 0 days (IQR 0–3)) than treated exacerbations (3 days (IQR 0 – 6)) (p = 0.030). Figure 4 shows that only 40.9% of untreated exacerbations were associated with a fall in daily step count between baseline and exacerbation compared to 68.4% for treated exacerbations (p = 0.025).

## Discussion

This is the first and largest study to report fall and recovery in daily walking activity using pedometry in ambulatory COPD patients during non-hospitalized exacerbations. We found that daily step count fell by 480 steps per day during exacerbation and recovery took a median 3.5 days. Over time, daily step count fell faster in patients with frequent exacerbations. We also observed that exacerbations associated with a fall in daily step count were more likely to be reported and received treatment.

Previous work on activity during exacerbation recovery has been mainly based on the study of hospitalised COPD patients. Pitta and colleagues saw improvements in time spent walking in hospital compared to one month after discharge, but they observed no difference between day 2 and day 7 [11]. Borges and colleagues have shown that daily step count assessed with a tri-axial accelerometer increased from 602 steps/day on the 2^nd^ day of hospitalisation to 3,575 steps/day at one month post discharge (p < 0.001) in 32 patients [10]. However, activity monitoring during exacerbations that required hospitalisations may not reflect patient behaviour during exacerbations taking place in the community as hospitalised patients are likely to stay in bed for prolonged periods and not undertake their usual activities. Furthermore, these hospital based studies did not collect data prior to the onset of the exacerbation. In a recently published small study, Ehsan reported on activity levels in 17 patients who had just 27 symptom-define (EXACT) exacerbation in the community [12] but daily data was lacking.

In this study we found that respiratory symptoms took longer than daily step-count or time outdoors to return to baseline values. The reasons for an earlier recovery in activity are unclear, but may be due to the need to go out of the home for social reasons or shopping, perhaps to return to work or a desire to exercise after a period of being housebound. It is also possible that the severity of the respiratory symptoms during the later stages of recovery is not sufficient to inhibit activity. In a previous study, on 1465 exacerbations, we reported that patients are significantly less likely to leave their home during an exacerbation [7]. In this study, we observed a similar trend, but it did not reach statistical significance due to the smaller patient numbers studied. As daily step count fell but hour’s outdoors was unchanged relative to the baseline, it might be that patients with an exacerbation used transport or walked more slowly when outside the home. The actual intensity and nature of exercise undertaken is a question that can only be addressed by more sophisticated accelerometer devices.

The present study shows that the larger the fall in the daily step count at exacerbation the longer it takes activity to return to normal. This suggests that COPD patients should be encouraged to keep active during the early stages of exacerbation since activity will return to normal faster. Pulmonary rehabilitation programmes initiated early post-exacerbation show a benefit compared to usual care [15-17,26-28] possibly by avoiding muscle deconditioning through inactivity during bed-rest [29]. However, in the largest study to date, Eaton and colleagues found no significant effect on acute health-care utilization, although it was found to be safe and feasible [30].

Treated exacerbations were slower to recovery compared to untreated exacerbations though our previous work has suggested there is little difference in respiratory symptoms between treated and untreated events [4]. The reasons why patients report some exacerbations to a health care professional and not others have been unclear and approximately 50% of exacerbations are unreported [4,31]. Our findings suggest that impairment of daily activity may be a key factor in patients seeking additional therapy for their exacerbation.

This study has shown for the first time that patients who experienced frequent exacerbations have twice as fast an annual decline in daily step-count than patients with infrequent exacerbations. Frequent exacerbations are known to accelerate both decline in FEV_1_[25] and rise in airway and systemic inflammation [3,32] with patients becoming housebound faster [7], and more likely to suffer from depression [33] and fatigue [34]. The precise mechanism of activity decline is unclear but it is possible that prolonged recovery or non-recovery after an exacerbation may contribute to this decline. In addition, anxiety, depression and fatigue are associated with exacerbations and any discourage patients from maintaining activity. This may be particularly marked in more severe patients with frequent exacerbations as they have significantly more and longer hospital admissions, though hospital admissions were not a subject for this study. Cote and colleagues found a reduction in exercise capacity after an exacerbation, with a 72 m decline (20%) in 6MWD [35].

An important strength of the study was the prospective daily monitoring of COPD patients to capture a baseline activity level just before the exacerbation event started. Baseline data collected away from the exacerbation may not be valid as daily step count declines over time and varies with seasonal changes [14]. Daily monitoring is also essential when investigating exacerbation where symptoms recovery to normal in a few days. One limitation of the study is that we did not include patients using ambulatory oxygen or walking supports or mild patients with GOLD stage 1 COPD and our results should not be extrapolated to these patient groups. However an important feature of this study is that exacerbations treated in the community were included and this makes the data applicable to the majority of exacerbations experienced by COPD patients.

## Conclusions

Pedometer measurements can be used to track daily activity easily during COPD exacerbations that do not require hospital admission. We have shown that exacerbations reduce physical activity with patients recovering within a 3 to 4 day period. Frequent exacerbations also hasten a decline in activity over time and potentially this patient group would benefit from greater encouragement to continue exercising. Our results also show that daily activity is a major drive for patients reporting exacerbation events and seeking additional therapy. Thus, non-hospitalized COPD exacerbations are key events that not only cause symptomatic deterioration but also impair the patients’ activity.

## Abbreviations

COPD: Chronic Obstructive Pulmonary Disease; PEF: Peak expiratory flow; PR: Pulmonary rehabilitation; FEV1: Forced expiratory volume in one second; FVC: Forced vital capacity; GOLD: Global initiative for chronic obstructive lung disease; SEM: Standard error of the mean; IQR: Inter quartile range; SD: Standard deviation.

## Competing interests

The authors declare that they have no competing interests.

## Authors’ contribution

AA, JW, GD designed the study and analyzed the data; AA, AP, BK, AM, RS, saw patients in clinic and collected data; AA, AP, BK, AM, RS, JW and GD contributed to interpretation and drafting the manuscript for important intellectual content. All authors read and approved the final manuscript.

## Pre-publication history

The pre-publication history for this paper can be accessed here:

http://www.biomedcentral.com/1471-2466/14/98/prepub

## Supplementary Material

Additional file 1 Characteristics of the 37 COPD patients in whom pedometry data was recorded during at least one exacerbation. Click here for file
