# Supplementary material for: Daily activity during stability and exacerbation of chronic obstructive pulmonary disease
Source: BMC Pulm Med. 2014 Jun 2;14:98. doi: 10.1186/1471-2466-14-98 (PMC4057595; doi:10.1186/1471-2466-14-98)
Supplement: Additional file 1 — Characteristics of the 37 COPD patients in whom pedometry data was recorded during at least one exacerbation. [file 1471-2466-14-98-S1.pdf]

Additional table (file 1): Characteristics of the 37 COPD patients in whom pedometry data was recorded during at least one exacerbation

	Mean	*(±SD)*
**Age (years)**	70.4	(±7.9)
**FEV_1 (l)**	1.4	(±0.5)
**FEV_1 (% predicted)**	52.1	(±15.7)
**FVC (l)**	3.0	(±0.8)
**FEV_1/FVC (%)**	46.2	(±13.3)
**BMI (kg/m^2)**	27.3	(±6.0)
	Median	***(IQR)***
**Exacerbations per year**	3	(1-3)
	**%**	
**Sex (Males)**	78.4	
**Smoking at recruitment**	32.4	
